# Overview of systematic reviews: Management of common Traumatic Brain Injury-related complications

**DOI:** 10.1371/journal.pone.0273998

**Published:** 2022-09-01

**Authors:** Vandana Vasudevan, Bhasker Amatya, Fary Khan

**Affiliations:** 1 Department of Rehabilitation, Royal Melbourne Hospital, Parkville, Victoria, Australia; 2 Australian Rehabilitation Research Centre, Royal Melbourne Hospital, Parkville, Victoria, Australia; 3 Department of Medicine, University of Melbourne, Parkville, Victoria, Australia; Universidade de Sorocaba, BRAZIL

## Abstract

**Background:**

Many clinical interventions are trialled to manage medical complications following Traumatic Brain Injury (TBI). However, published evidence for the effects of those clinical interventions is limited. This article is an overview of common complications and their management from published systematic reviews in TBI.

**Methods and findings:**

A health science electronic database search for published systematic reviews for management of common complications in TBI was conducted in the last decade till 31^st^ January 2021. Methodological quality and evidence were critically appraised using the Grading of Recommendations, Assessment, Development and Evaluations and Revised-Assessment of Multiple Systematic review tools. Overall, only six systematic reviews complied with search criteria, these evaluated fatigue, spasticity and post traumatic seizures (29 RCTs, 13 cohort studies, n = 5639 participants). No systematic reviews for other common TBI-related complications met criteria for this review. The included reviews varied from ‘moderate to high’ in methodological quality. The findings suggest beneficial treatment effect of anti-epileptic drugs (phenytoin/levetiracetam) compared with placebo in reducing early seizure incidence, but no significant benefit of phenytoin over levetiracetam, valproate, or neuroprotective agent for early or late posttraumatic seizures. There was ‘limited’ evidence for spasticity-related interventions, and ‘insufficient’ evidence of cardiorespiratory training on fatigue levels.

**Conclusions:**

Despite the high prevalence and associated functional impact of TBI-related complications, there is limited evidence to guide treating clinicians for management of common TBI complications. More robust studies are needed to build evidence in this population.

## Introduction

Traumatic Brain Injury (TBI) is a global health problem and leading cause of death and disability worldwide. It is defined as “an alteration in brain function, or other evidence of brain pathology, caused by an external force” and may include a change in the mental status, loss of consciousness, focal neurological deficit, or loss of memory for the event [1]. Based on the extent of damage to the brain and its severity, TBI can be classified into mild, moderate and severe. High incidence of TBI is found amongst young people (15–35 years) and is more common in men (male: female ratio = 3–4:1) due to risk-taking behaviour [2]. The estimated rate of TBI incidence globally is varied, and is between 100 and 300 per 100,000 population, with mild TBI accounting for 70% to 90% of all TBIs [3, 4]. The incidence rate of TBI in USA in 2016 was 333 per 100,000 population compared to Australia with 275 per 100, 000 population [3]. The estimated lifetime cost per incident for severe TBI was approximately US$ 4.8 million [5]. Globally, 10 million people are affected annually, making it an important public health issue [6].

Advances in medical and intensive management have reduced mortality rates for people with severe TBI, but the burden of care remains substantive. TBI can result in multiple complications, the sequelae of which can vary in their nature and severity. These issues can have a significant impact on the daily function with disabilities (e.g.: mobility, activities of daily living, fatigue, pain) and participation (e.g., quality of life). Motor, cognitive, behavioural and personality deficits, which may occur following TBI, can be disabling. Some potential common complications, especially after moderate-severe TBI include posttraumatic seizures (PTS) and epilepsy, hydrocephalus, paroxysmal sympathetic hyperactivity, spasticity, agitation, neuroendocrine dysfunction, heterotrophic ossification, venous thromboembolism, sleep disturbances and cranial nerve dysfunction [5, 7–10]. The reported incidence of posttraumatic seizures and epilepsy vary between 4–53% [10]. Depending on the severity of TBI, the propensity of seizure development in TBI survivors is 1.5–17 times more than the general population [10, 11]. Spasticity, is common after moderate to severe TBI, and can limit mobility and independence with daily activities. However, there is limited epidemiological data for spasticity in TBI. Pharmacological and non-pharmacological interventions, often in combination are used to manage spasticity [12]. Fatigue is also common following TBI and can occur in 33–64% of TBI victims [13, 14].

Autonomic dysfunction is well documented in TBI survivors, with a reported incidence of 8 to 32% [10]. Studies show that approximately 30% of moderate to severe TBI patients develop hypopituitarism over the first year after injury [11]. Growth hormone (GH) is the most commonly deficient hormone although thyroid-stimulating hormone (TSH) and cortisol levels can also be abnormal after brain injury [10]. Sleep disorders are a well-established complication with approximately 30–50% of patients complaining of new-onset or worsening insomnia after TBI [15]. TBI survivors with disrupted sleep-wake patterns have longer durations of rehabilitation, exacerbated cognitive dysfunction and poorer long-term vocational outcomes [16, 17].

TBI complications are complex and multi-faceted, and often require a comprehensive approach using pharmacological and/or non-pharmacological interventions. Rehabilitation following TBI is best managed by a specialised interdisciplinary approach, with goals of minimising complications and optimising function using adaptive environmental modifications. To date, many clinical trials evaluating TBI complications have been conducted and numerous systematic reviews have been published compiling the evidence from these primary studies for the management of TBI complications. However, they differ in their scope, quality and methodology. An overview of published systematic reviews is thus required to qualitatively appraise the methodology and to systematically evaluate evidence for the management of potential medical complications following TBI. Therefore, this article provides an evidence-based overview of the management of common TBI-related medical complications from published systematic reviews of clinical trials. It is envisaged that the findings will provide an evidence-based snapshot to guide treating clinicians and also for future research by highlighting existing gaps in evidence.

## Methods

The review was reported in accordance with the Preferred Reporting Items for Systematic Reviews and Meta-Analyses (PRISMA) (S1 Appendix) and Cochrane Library Handbook [18]. A comprehensive search of the Cochrane Library Database, MEDLINE, PubMed, and Embase was undertaken for the last ten years till January 31, 2021, for systematic reviews evaluating various management approaches for TBI-related complications. Most of the relevant qualitative studies were in the last ten years and any search beyond this is unlikely to add any clinically relevant information to guide evidence-based practice. The search was constructed in Ovid using a combination of multiple search items for 3 themes: ‘traumatic brain injury’ ‘systematic review/meta-analysis’ and ‘complication or complications’ (S2 and S3 Appendices). A combination of MeSH terms and keywords were used to search other databases. A manual search of the relevant bibliographies of potential articles and relevant journals and a grey literature search using different Internet search engines and websites were undertaken. All systematic reviews/meta-analyses that evaluated pharmacological/non-pharmacological intervention for the management of TBI-related medical complications, English publications and adult population (age over 18 years) were included in this review. The exclusion criteria include: non-English language publications, narrative reviews, paediatric population, reviews with studies that do not provide separate data for TBI cohorts and those with the acquired non-TBI population. Also, systematic reviews on cognitive and behaviour management following TBI have been extensively published and were excluded for the purpose of this review.

### Study selection and data extraction

Two reviewers (VV, BA) screened and shortlisted all abstracts and titles of review identified by the search strategy based on the search criteria. All studies identified through the search process were exported to an EndNote X9 (Clarivate, London, UK) database for the removal of duplicates. The authors then evaluated each study independently, and the full text of all potential articles was assessed to determine eligibility based on the inclusion/exclusion criteria. Any disagreement regarding the eligibility criteria was discussed with a third reviewer (FK) and resolved by a final group consensus. Standard proforma was used for data extraction from all reviews, which included publication and search date, objectives, characteristics of included studies and study subjects, interventions, findings/patient outcomes in the review, and limitations.

### Assessment of methodological quality

#### Quality of included reviews

Two authors (VV, BA) independently assessed the methodological quality of each review, using the Revised-Assessment of Multiple Systematic reviews (R-AMSTAR) appraisal tool [19] (S4 Appendix). Any disagreements were resolved by consensus among all authors.

The R-AMSTAR consists of 11 questions for methodological quality assessment of systematic reviews [20, 21]. It assesses the quality of systematic reviews with an acceptable inter-rater agreement, construct validity and feasibility. A score of 1 to 4 was given to each domain based on predefined criteria for each item (yes: considered adequate, or no: considered inadequate). The overall methodological quality of each review was calculated by the sum of total scores, which ranged from 11 to 44. For this overview, scores of 40 or higher were considered of high quality; scores of 30–39 of medium quality, and scores below 29 of low quality [22].

#### Quality of evidence of included reviews

The Grading of Recommendations, Assessment, Development and Evaluations (GRADE) system [23] on a four-point rating scale (very low, low, moderate and high quality) assessed the quality of evidence for each type of outcome. An a priori ranking was assigned: “high” for randomised controlled trials (RCTs) and “low” for non-RCTs. Based on the author’s judgement, initial grading of the included review was either up or downgraded. The review was downgraded mainly due to the presence of risk of bias, inconsistency, indirectness of evidence or imprecision of the publication bias; or upgraded because of large effect size and consistency in the findings. Any disagreements were resolved by a final group consensus amongst all authors.

## Results

**Fig 1:**
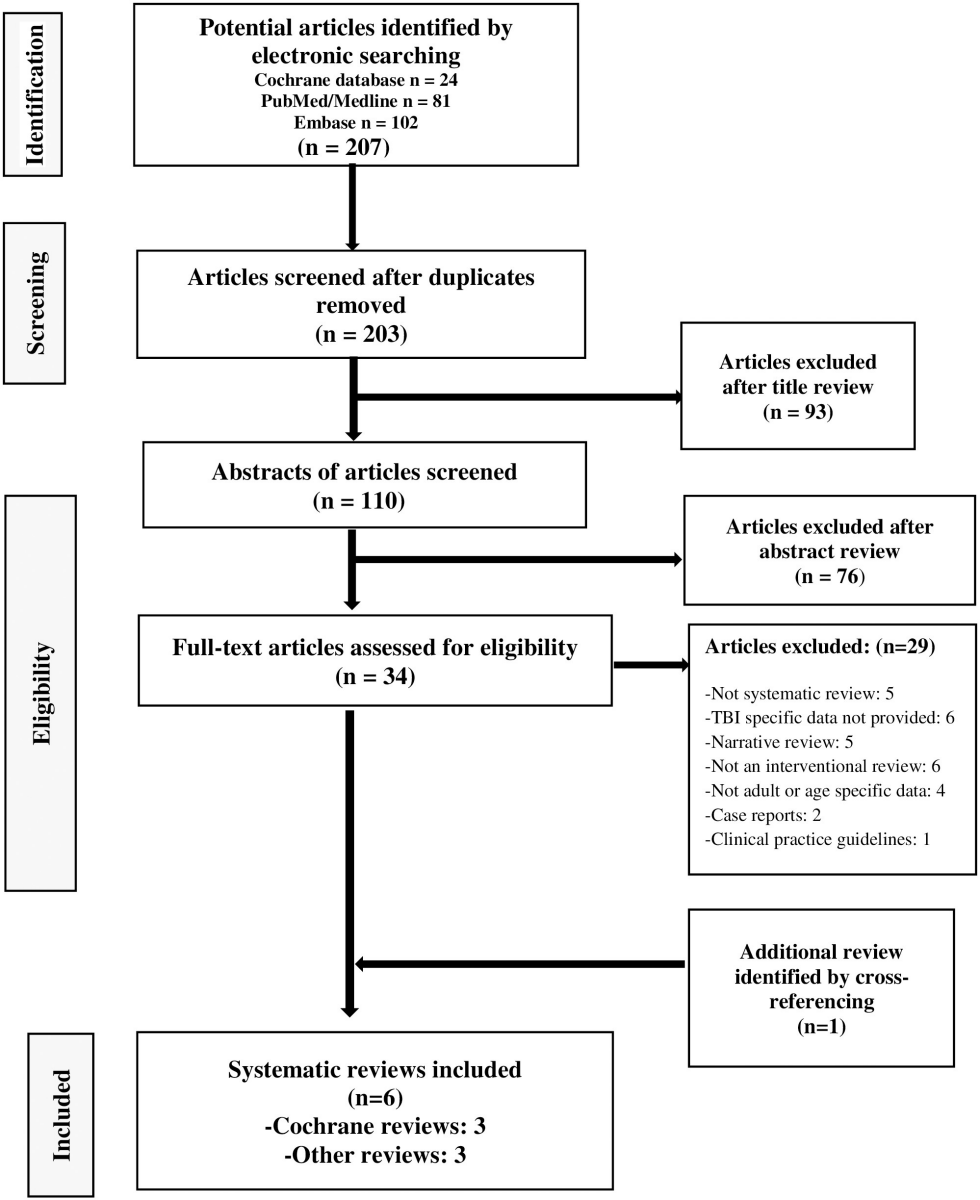
PRISMA flow diagram showing selection of reviews.

The initial search identified a total of 207 published systematic reviews (Cochrane database = 24, PubMed/MEDLINE = 81 and Embase = 102) evaluating the management of potential common medical complications following TBI. Of these, 34 met the abstract inclusion criteria and were selected for closer scrutiny. One review was identified from the manual search of the bibliography of relevant articles. Overall, six reviews evaluating fatigue, spasticity and post traumatic seizures were included, (a total of 29 RCTs and 13 cohort studies with 5639 participants). Of these, three reviews were published in the Cochrane Library database and three in other academic journals. Despite the high clinical relevance and common occurrence of other TBI- related complications, no systematic reviews addressing them were identified or met standard selection criteria of this review. A PRISMA flowchart of the study selection process is provided in Fig 1. Detail of excluded reviews with reasons for exclusion is provided in S5 Appendix.

### Quality assessment of the included reviews

The summary results of the R-AMSTAR quality assessment are provided in Table 1 with a detailed assessment score for each item in S4 Appendix. The overall mean R-AMSTAR methodology quality score for included reviews was 37.8±5.0 (range 30–42) out of a maximum score of 44. The quality of included reviews varied from moderate to high, of which 3 Cochrane reviews [24–26] were of high quality (i.e., total score = 42), whilst the remaining 3 were of moderate quality (total score = 30–36) [27–29]. For the purpose of this review, cognitive and behavioural complications were excluded as mentioned above, as these topics have been published extensively. All included reviews searched at least two medical science databases; however, the non-Cochrane reviews were not supplemented by other search criteria mentioned in the R-AMSTAR tool. *A priori* protocol publication criteria were met for reviews, except for two [28, 29]. All included reviews, except one [27] had at least two independent reviewers for study selection and data extraction. All three Cochrane reviews [24–26] provided details of the included and excluded studies and met the criteria for grey literature search. The scientific quality of all primary studies included in these reviews were assessed using different validated tools such as Physiotherapy Evidence Database (PEDro) scale, Newcastle-Ottawa Quality Assessment Scale, Jadad scale and Cochrane Risk of Bias Tool. Although all included reviews provided clear details of the characteristics of primary studies (and funding sources), none addressed the author’s competing interests in the primary studies.

**Table 1:** Quality assessment (R-AMSTAR) * of included systematic reviews.

R-AMSTAR Criteria*	Author, year					
	Hassett 2017	Synnot 2017	Thompson 2015	Bakr 2018	Meshkini 2015	Khan 2016
1.	4	4	4	4	3	3
2.	4	4	4	1	4	4
3.	4	4	4	3	3	3
4.	4	4	4	2	2	2
5.	4	4	4	2	1	2
6.	4	4	4	4	4	4
7.	4	4	4	4	4	4
8.	4	4	4	4	3	4
9.	4	4	4	2	4	4
10.	3	3	3	1	4	3
11.	3	3	3	3	3	3
**TOTAL** (Out of 44)**	**42**	**42**	**42**	**30**	**35**	**36**

* Revised-Assessment of Multiple Systematic reviews (R-AMSTAR) appraisal tool (19), details please refer to S4 Appendix.

**R-AMSTAR cut–off scores: High quality ≥40; Medium quality 30–39; Low quality ≤29

### Evidence synthesis of interventions for common TBI medical complications

The evidence for all relevant outcomes with the GRADE rating is summarised in Table 2.

**Table 2:** Summary of evidence for common TBI medical complications.

Author, year	Complication evaluated	Intervention	Included studies	Participants	Main results/findings	Quality of evidence (GRADE)
**Hassett et al, 2017**	Fatigue	Cardiorespiratory fitness training	8 RCTs Search date: up to Aug 2017 (update) **Meta-analysis: Yes**	399 with all TBI severity types	Insufficient evidence for the effect of cardiorespiratory fitness training alone on fatigue compared to usual care, non-exercise intervention or no intervention (*SMD-0*.*32*, *95% CI-0*.*90 to 0*.*26)*	Very low
**Synnot et al, 2017**	Spasticity	*Pharmacological (*intrathecal baclofen*, *Botulinum toxin*) and non-pharmacological interventions (*casting*, *traditional splints*, *pseudo elastic orthosis*, *physiotherapy*) (often combined)	9 RCTs Search date: up to June 2017 **Meta-analysis: No**	134 with skeletal muscle spasticity following TBI	Low quality evidence for both pharmacological and non-pharmacological interventions, often in combination compared with placebo/no treatment for upper and lower limb skeletal muscle spasticity*	Very low
**Thompson et al, 2015**	Post-traumatic epilepsy (PTE)	i) Antiepileptic drugs (AEDs) (*Phenytoin*, *Carbamazepine)* vs placebo or standard care ii)Neuroprotective agent (*Magnesium sulfate*) vs placebo iii) Phenytoin vs AEDs (*Levetiracetam*, *Valproate)*	10 RCTs Search date: up to January 2015 **Meta-analysis: No**	2326 participants with moderate and severe TBI	Low quality evidence for early treatment with an AED in reducing risk of early post-traumatic seizures compared with placebo or standard care *(RR 0*.*42; 95% CI 0*.*23 to 0*.*73)* No evidence in the risk of late seizure occurrence *(RR 0*.*91; 95% CI 0*.*57 to 1*.*46)* or mortality (*RR 1*.*08*; 95% CI 0.79 to 1.46)) between AEDs and placebo or standard care Insufficient evidence for neuroprotective agents compared to placebo *(RR 1*,*07; 95% CI 0*.*53 to 2*.*17)* Insufficient evidence for effectiveness or safety of phenytoin with another AED (levetiracetam, valproate) in reducing early *(RR 0*.*66; 95% CI 0*.*20 to 2*.*12;)* or late seizures (*RR 0*.*77; 95% CI 0*.*46 to 1*.*30;)*	Low Very low Low Low-moderate
**Bakr et al, 2018**	Late post- traumatic seizures	Levetiracetam and phenytoin	1 RCT and 1 cohort study Search date: up to April 2016 **Meta-analysis: No**	71 participants with mild to severe TBI	No difference in late seizure incidence or length of hospital stay between levetiracetam and phenytoin (p > 0.055 for both) Significant improvement in GOS-E with levetiracetam at 6 months *(p = 0*.*016; 95% CI -1*.*0 to -8*.*5)*	Very low
**Meshkini et al, 2015**	Post-traumatic seizures	Levetiracetam and phenytoin	6 cohort studies Search date: up to November 2014 **Meta-analysis: Yes**	1523 participants with TBI	Equal efficacy in seizure prevention between levetiracetam and phenytoin *(OR = 1*.*1; 95% CI 0*.*55–2*.*20*,)	Low
**Khan et al, 2016**	Post-traumatic seizures	Levetiracetam and phenytoin	1 RCT and 6 cohort studies Search date: up to June 2015 **Meta-analysis: Yes**	1186 participants with severe TBI	No difference in the incidence of early seizures after TBI with either levetiracetam or phenytoin *(RR 1*.*02*, *95% CI 0*.*53–1*.*95*, *p = 0*.*96)*	Low

*Refer to the primary review [24] as multiple interventions were evaluated

AEDs: Antiepileptic drugs, GOS-E: Extended Glasgow Outcome Scale, GRADE: Grading of Recommendations, Assessment, Development and Evaluations and Revised-Assessment of Multiple Systematic, OR: Odds Ratio, RR: Risk Ratio, RCT: Randomised Controlled Trial, SMD: Standardised Mean Difference, 95% CI: 95% Confidence Interval, TBI: Traumatic Brain Injury

#### Fatigue

One systematic review (8 RCTs, n = 399 participants) [25] evaluated the effectiveness of an exercise program incorporating cardiorespiratory fitness training (primary outcome) following TBI of any severity compared with usual care, a non-exercise intervention, or no intervention. The experimental intervention was implemented in different settings, (inpatient, and ambulatory). Fatigue was measured as a secondary outcome measure. Majority of the participants were male (71%) with severe TBI (58%), with an average age of 35 years. The time from injury ranged from 1.3 to 40.8 months. The intervention included exercises using large muscle groups prescribed at least 3 times a week for at least 20 minutes for 4–12 weeks. Of the eight included primary studies, only three (n = 130 participants) [30–32] evaluated fatigue as a secondary outcome, but used different tools: Visual Analogue Scale (VAS), fatigue subscale of Profile of Moods State and modified version of Chalder Fatigue Scale. Although fitness training showed a small reduction in fatigue levels on VAS compared to control interventions, this was not statistically significant (Standardised Mean Difference (SMD) = -0.32, 95% Confidence Interval (CI) = -0.90 to 0.26).

#### Spasticity

Synnot et al. (9 RCTs, n = 134 participants) [24] investigated the effects of a range of pharmacological and non-pharmacological interventions, for skeletal muscle spasticity after TBI. Of the nine included studies, only five (n = 105 participants) provided between-group differences that contributed outcome data to the results of this review. These five trials assessed various interventions, often in combination, for spasticity management. Majority of the participants were male (60%-92%) with ages ranging from 24 years to 41.5 years. Spasticity was assessed using different scales such as Tardieu scale, Ashworth Scale or the Modified Ashworth Scale [24].

*Pharmacological treatments*. Pharmacological interventions evaluated within the review included intrathecal baclofen [33] and botulinum toxin A (BoNT-A) [34, 35]. Only one RCT by Meythaler et al. (n = 11 participants) [33], compared the effect of intrathecal baclofen (50mcg) versus saline placebo for spasticity in the upper and lower limbs in an outpatient setting. The authors reported a significant improvement in spasticity with intrathecal baclofen (50mcg) compared to placebo after four (p = 0.0084) and six (p = 0.0163) hours of treatment in the lower limbs. Similarly, the results were reported as significant with baclofen compared to placebo in the upper extremity at four hours after administration (p = 0.0097). However, this effect was not sustained at six hours (p value not reported). No adverse effects were reported in either group.

Two studies compared the effect of BoNT-A versus placebo (with or without casting) for upper and lower limb spasticity for TBI survivors in either an acute or sub-acute setting [34, 35]. The doses of BoNT-A used in studies ranged from 200U to 1000U [34, 35]. Although Gracies et al. (n = 23 participants) [34] reported a greater beneficial effect for the BoNT-A compared with placebo in improving upper limb (elbow, wrist and finger flexors) spasticity at four weeks after treatment, the details of outcome data were not provided. Another RCT (n = 25 participants) [35] failed to demonstrate any beneficial effect of BoNT-A for calf muscle spasticity (with casting) compared to placebo plus casting (Mean Difference: MD 0.30; 95% CI -0.87 to 1.47) at 12 weeks post-intervention. The most common adverse event reported was mild muscle weakness. BoNT-A was well tolerated except for one participant reporting flu-like symptoms.

*Non-pharmacological treatments*. Non-pharmacological interventions evaluated within the review included: casting, physiotherapy [35], splinting [36], electrical stimulation and tilt tabling [37]. One RCT [37] evaluated the effectiveness of a multi-modal treatment involving tilt table standing (30 minutes) with electrical stimulation to ankle dorsiflexors (5 times/week) with ankle splinting (12 hours/day) for at least five days a week compared with the control group receiving tilt table standing (30 minutes, 3 times/week) only. The authors reported a small mean reduction in spasticity at week six favouring the intervention group (MD -1.00; 95% CI 0.1 to 1.8), which disappeared at end of the trial (10 weeks). No adverse events were reported.

Verplancke et al. [35] in another multi-group RCT (n = 11 participants) compared physiotherapy (PT) alone versus placebo and casting or BoNT-A and casting at 12 weeks for lower limb spasticity. The findings suggested no significant differences between both treatments (PT vs. placebo: MD -0.80; 95% CI -2.00 to 0.40; PT vs. BoNT-A: MD -0.50; 95% CI -1.82 to 0.82). One serious adverse event each was reported in the PT group (deep vein thrombosis) and placebo group (joint contracture).

Another RCT (n = 25 participants) [36] evaluated pseudo-elastic orthosis compared with a traditional (static) splint for ankle or elbow spasticity. The authors found no improvement in upper and lower limb spasticity at one-month post-intervention (P value not reported).

#### Posttraumatic seizures (PTS)

One systematic review (10 RCTs, n = 2326 participants) [26] compared the efficacy of antiepileptic drugs (AEDs) and neuroprotective agents with placebo or usual care for PTS prophylaxis in patients with moderate to severe TBI. Five trials in this review that compared the treatment of a traditional AED (phenytoin or carbamazepine) with placebo or usual care showed a significant reduction in risk of early seizure incidence in the intervention group (Relative Risk (RR): 0.42, 95% CI: 0.23 to 0.73). Duration of the treatment with an AED varied between five to seven days. Regarding late seizure incidence, the authors reported a non-statistically significant effect for use of AEDs compared with placebo or usual care (RR: 0.91, 95% CI 0.57 to 1.467). The treatment duration varied from three months to three years. Two RCTs [38, 39] comparing phenytoin with another AED (levetiracetam or valproate) showed no statistically significant treatment benefit of phenytoin over the other AEDs for early (RR: 0.66, 95% CI: 0.20 to 2.12) or late seizures (RR: 0.77, 95% CI: 0.46 to 1.30). Another RCT [40] evaluating the efficacy of a neuroprotective agent (magnesium sulfate) with a placebo did not report any data for early seizure occurrence. Further, there was no beneficial effect of neuroprotective agents compared with placebo for late seizure occurrence (RR: 1.07, 95% CI: 0.53 to 2.17). Another systematic review (1 RCT and 6 cohort studies, n = 1186 participants) [28], investigated the effectiveness of levetiracetam compared with phenytoin for seizure prophylaxis in patients with severe TBI. Five studies used electroencephalography to describe a seizure, and most studies conducted follow-up for up to 7 days, therefore analysis was only performed for early seizure risk occurrence. The reviewers reported no statistically significant group difference for early seizure prophylaxis (RR: 1.02; 95% CI: 0.53–1.95; p = 0.96). Adverse drug events were more frequent in the phenytoin group compared with the levetiracetam group (13 vs. 7%).

Another meta-analysis (6 cohort studies, n = 1523 participants) [29] assessed the effectiveness of phenytoin versus levetiracetam for seizure prophylaxis in patients with TBI. The occurrence of seizure was the primary outcome measure and assessed at intervals (7 days to 30 months). The authors report no significant group difference between the evaluated AEDs in post-traumatic seizure prophylaxis (Odds Ratio (OR) = 1.1, 95% CI: 0.55–2.20). The rates of complications in TBI patients treated with levetiracetam were fewer than those treated with phenytoin.

Bakr et al. in another systematic review (1 RCT, n = 52 participants; and 1 cohort study, n = 19 participants) [27] evaluated the efficacy of phenytoin and levetiracetam in the prevention of late post-traumatic seizures following severe TBI. Secondary outcomes were the length of hospital stay and the Extended Glasgow Outcome Scale (GOS-E) at 6 months. The duration of prophylactic treatment with an AED in both trials was seven days. Both studies concluded that phenytoin and levetiracetam were equivalent in their efficacy for prevention of late post-traumatic seizures (for RCT, p = 1.0, 95% CI: 0.263 to 9.082; for cohort, p = 0.53, 95% CI: 0.191 to 3.690). Further, both studies did not report any significant difference in the length of stay (p > 0.05 for both). However, the RCT showed a significant improvement in both the GOS-E (p = 0.016, 95% CI: -1.0 to -8.5) and the disability rating scale (DRS) (p value not provided) with levetiracetam at six months.

In summary, three systematic reviews [27–29] evaluated the efficacy of phenytoin and levetiracetam in post-traumatic seizure prophylaxis and safety. The studies reported no statistically significant group difference between them for early and late seizure occurrence. However, the complication rates and adverse drug events were found to be more frequent in the phenytoin group. Additionally, Bakr et al, in his systematic review did not find any significant difference in the length of hospital stay, which was evaluated as a secondary outcome measure. However, a significant improvement in the GOS-E and DRS were noted at six months in the levetiracetam group.

## Discussion

This review systematically summarizes the up-to-date evidence from published systematic reviews to provide an overview of the interventions currently used for the management of common medical complications following mild to severe TBI. An overview of systematic reviews is an emerging method of appraising and synthesising evidence from published systematic reviews on similar or related topics. It can be a useful tool to summarise up-to-date information on treatment effects of the same or similar interventions in a much broader concept to guide and support decision-making by clinicians, policymakers and clinical guideline developers. Overall, only six systematic reviews met the inclusion criteria and were included in this review. These evaluated three common complications: posttraumatic seizures, fatigue and spasticity. The findings from this review indicate that despite a wide range of treatment modalities for the management of these three complications, the evidence to support them is low or insufficient. Further, this review identified paucity of data from published systematic reviews addressing management of other common complications related to TBI, due to the limited number of methodologically robust studies.

The key findings of this review, based on GRADE approach suggest:

### Posttraumatic seizures

- *Moderate quality* evidence for the effectiveness of phenytoin with another AED (valproate, levetiracetam) in reducing early or late seizures [26]
- *Low-quality* evidence for AEDs (phenytoin, carbamazepine) in reducing the risk of early seizures compared with placebo or standard care [26].
- *Low-quality* evidence for the efficacy of levetiracetam or phenytoin in the incidence of early seizure prophylaxis [28, 29]
- *Low-quality* evidence for neuroprotective agent (magnesium sulfate) compared with placebo in seizure prophylaxis following moderate-severe brain injury [26]
- *Low-quality* evidence for safety or effectiveness of phenytoin compared with another AED (valproate, levetiracetam) in reducing early or late seizures [26]
- *Very low-quality* evidence for AEDs (phenytoin, carbamazepine) in reducing the risk of late seizures compared with placebo or standard care [26]
- *Very low-quality* evidence for either phenytoin or levetiracetam for late seizure incidence risk, length of hospital stays or for levetiracetam on the long-term neurological outcomes (GOS-E scores) at six months following severe TBI [27]

Antiepileptic drugs (AEDs) are recommended for the treatment of early and late posttraumatic seizures. Historically, phenytoin has been the drug of choice for PTS prophylaxis following various brain injuries, including TBI. Although the efficacy of phenytoin has been widely accepted, its side effects remain a significant problem, especially longer-term use [41]. Hence, levetiracetam, a novel AED, with a better safety profile, is an alternative for PTS prophylaxis. Other studies [41–44] comparing the efficacy and safety between phenytoin and levetiracetam for seizure prophylaxis in brain injured patients show diverse results. While one meta-analysis by Zafar et al. showed similar efficacy for both AEDs in seizure prevention following brain injury [41], interestingly, other two meta-analyses evaluating phenytoin and levetiracetam for PTS prophylaxis in brain injury found effectiveness and better safety profile of levetiracetam over phenytoin [43, 44]. The authors reported that findings were possibly related to the variable dosing regimens, heterogeneous patient cohorts and different pharmacokinetic profiles between the groups. Further, several other studies in TBI population report no significant differences between phenytoin and levetiracetam in early/late seizure occurrence [42, 45, 46]. Despite the results from these studies being consistent with the findings of this review, they did not provide any TBI sub-group specific data or meet the inclusion criteria of this review, and hence were excluded.

Based on the current evaluation, AEDs are effective in early seizure prophylaxis and do not appear to alter the natural history of the late seizures or PTE. A recent meta-analysis by Wat et al. [47] highlighted the limited evidence available for use of other AEDs (carbamazepine, valproate) compared to placebo or no treatment in early seizure prophylaxis. Phenytoin and levetiracetam are equally effective in early PTS prophylaxis, however more robust studies are required to assess the efficacy of other AEDs in seizure prophylaxis. Whilst phenytoin use in early PTS has been researched widely, one should consider whether the overall benefit outweighs the complications associated with the use of this treatment. In cohort studies, levetiracetam appears to have a better safety profile compared to phenytoin, however, this has to be further confirmed in well-defined RCTs. The current review did not find any additional benefit in continuing seizure prophylaxis beyond the first week for late PTS prevention. This is also consistent with the most recent Brain Trauma Foundation Guidelines [48]. A single RCT [40] evaluating the neuroprotective effect of magnesium sulfate showed limited evidence for early and late seizure prophylaxis and hence warrants further high-quality prospective studies to justify its use in clinical practice.

### Fatigue

- *Very low-quality* evidence for the beneficial effect of cardiorespiratory exercise program on fatigue compared to usual care, non-exercise intervention or no intervention [25]

Posttraumatic fatigue, similar to sleep disorders, is a well-described complication following TBI. It can be primary (central) or secondary, and can exacerbate other co-morbidities. It usually occurs in conjunction with sleep disturbances causing cognitive, emotional and physical impairments. However, there are limited systematic reviews evaluating fatigue in the TBI population. Despite numerous narrative reviews [49–52] providing insight into post–traumatic fatigue, there is limited evidence on the associations between fatigue and other clinical variables such as depression, sleep dysfunction, medical comorbidities, physical and other cognitive impairment, and their impact on outcomes following injury. Further, there are no specific scales to assess fatigue following TBI. Experts argue that commonly used scales such as the Visual Analogue Scale (VAS), Fatigue Severity Scale (FSS) and Global Fatigue index (GFI) may not be sensitive enough to capture the severity of fatigue and its effects on TBI survivors [13]. Although fatigue is a common, long-lasting problem following TBI, the variation in the findings highlights the heterogeneity of post-traumatic fatigue and the need for further research on this topic.

### Spasticity

- *Very low-quality* evidence for the effectiveness of pharmacological (BoNT-A, intrathecal baclofen) and non-pharmacological interventions (casting, splinting, electrical stimulation, tilt table standing, PT), often used in combination for the management of upper and lower limb spasticity [24]

Spasticity is a prevalent physical complication of TBI. This review found only one systematic review [24] that evaluated various pharmacological and non-pharmacological interventions for spasticity management in TBI, with mixed results, due to limited studies, small sample size and heterogeneous interventions. Other studies evaluating spasticity in neurological conditions, (including TBI), report outcomes with similar mixed results [12, 53]. A critical appraisal of Clinical Practice Guidelines (CPGs) for spasticity management in TBI by Pattuwage et al. [54] highlights the need for more TBI-specific spasticity CPGs given the treatment challenges in these cohorts. The measurement of spasticity, specifically in TBI patients, in clinical practice can be challenging, and the type of intervention, treatment, and dose/intensity have been debated [55]. This is consistent with the findings of this review, indicating a selection of various pharmacological and/or non-pharmacological treatment options for the clinicians. Further, most of the information for spasticity management are derived from stroke research, thus highlighting the scarcity of data in spasticity after TBI and the need for future research with translation into clinical practice [55].

### Other systemic complications

Systemic complications following TBI can affect a multitude of systems including cardiovascular, respiratory, immunological, haematological and neuroendocrine systems. They can occur in the acute setting and continue through to rehabilitation and longer-term. Although these complications are commonly encountered and managed in routine clinical practice, this review found scarcity of data from published systematic reviews. Over the last decade, sleep-wake disturbances/deficits after TBI have been widely studied [56–58]. A meta-analysis by Grima et al. [59] compared sleep in the community-dwelling TBI patients relative to a healthy, control population without TBI. The primary objective sleep outcome measures were derived from polysomnography whereas secondary outcomes were subjective sleep measures such as the Epworth Sleepiness Scale (ESS) and the Pittsburgh Sleep Quality Index (PSQI). The results showed poorer sleep efficiency, longer sleep onset latencies, shorter total sleep duration, greater wake after sleep onset time and a lower percentage of rapid eye movement (REM) sleep time in TBI individuals as compared to controls. Similarly, there was an increase in subjective sleepiness and poor perceived sleep quality in the TBI cohort, consistent with polysomnographic derangement. These findings are consistent with the current literature, thus highlighting the need for further monitoring and addressing sleep deficits in this population. However, only a relatively few treatments have been proven effective, and need more research. Posttraumatic hypopituitarism can result in several neuroendocrine conditions, including growth hormone, gonadotrophin deficiencies and hypothyroidism. Aimaretti et al. [60] reported that 5% of TBI patients with normal pituitary functioning at 3 months, develop deficits a year later post-injury, likely due to loss of pituitary neuronal reserve, consistent with other reviews [61–63]. Hence, screening and treatment for neuroendocrine dysfunction is needed in the acute and chronic phases of TBI. Olfactory nerve dysfunction is commonly reported cranial nerve dysfunction after TBI, followed by facial and vestibulocochlear nerves. A systematic review [64] evaluating post-TBI olfactory impairment highlighted its presence to be a potential marker of additional structural and functional morbidities. The clinical relevance of these complications and paucity of data from systematic reviews highlights the need for further high-quality clinical trials to guide clinical practice.

### Limitations

There are several limitations with regard to the methodology and completeness of this review. The search was limited to the last decade only as most of the relevant qualitative studies were in the last ten years and any search beyond this is unlikely to add any clinically relevant information to guide evidence-based practice. Despite using the multi-pronged search approach to identify relevant studies, we identified only published systematic reviews from the academic databases and no reviews were found in the grey literature search. Publication bias cannot be ruled out as we were unable to include unpublished studies and most of the included systematic reviews were not up to date, and may have missed many recent studies. Therefore, the findings of this review should be interpreted with caution. The included reviews assessed a small number of primary studies evaluating specific TBI-related outcomes, which may have an impact on the formulation of the level or quality of evidence to make any definitive conclusions about the interventions. The settings and measurement tools used in various reviews were different even for the same evaluated intervention, which precluded pooling of data. Further, the evaluation of original data from the primary studies within the included reviews was beyond the scope of this review as the primary objective was to analyse the findings from the included reviews themselves. Systematic reviews addressing other common TBI-related complications (neuroendocrine, haematological etc.) did not met the selection criteria of this review, and hence were excluded. A reference bias may have been introduced by scrutinising only the reference lists within the relevant articles. Although GRADE and R-AMSTAR tools are validated and widely used, they have certain limitations. It was beyond the scope of this review to evaluate the evidence on the safety of some of the included interventions. This review did not evaluate specific cognitive and behaviour therapies in TBI as this topic has already been extensively published [65–69] and was beyond the scope of this review.

## Conclusion

TBI is a complex injury with complications causing significant societal and financial burden to patients (and families), and health system at large. These TBI-related complications require comprehensive management using pharmacological and/or non-pharmacological approaches, including rehabilitation, and longer-term supportive care. Despite high prevalence of various complications in TBI population, evidence for many is lacking. The most common complications evaluated were posttraumatic seizures, fatigue and spasticity. Unfortunately, management of many of the other common TBI complications were not included given paucity of systematic reviews in this population. Larger robust trials are needed to build evidence for the effectiveness of various TBI-related complications to guide clinical practice.

## Supporting information

S1 Appendix PRISMA checklist. (DOC)

S2 Appendix EMBASE search strategy. (DOCX)

S3 Appendix Medline search strategy. (DOCX)

S4 Appendix Quality assessment (R-AMSTAR) of included systematic reviews. (DOCX)

S5 Appendix Details of excluded reviews with reason. (DOCX)

S6 Appendix References of excluded reviews. (DOCX)
