# Supplementary material for: Overview of systematic reviews: Management of common Traumatic Brain Injury-related complications
Source: PLoS One. 2022 Sep 1;17(9):e0273998. doi: 10.1371/journal.pone.0273998 (PMC9436148; doi:10.1371/journal.pone.0273998)
Supplement: S2 Appendix — (DOCX) [file pone.0273998.s002.docx]

**S 2 Appendix. EMBASE searched strategy**

| 1 | exp head injury/ |
| --- | --- |
| 2 | exp Cerebrovascular Accident/ |
| 3 | exp Brain Edema/ |
| 4 | "Glasgow coma scale".ab,ti. |
| 5 | "Glasgow outcome scale".ab,ti. |
| 6 | "Glasgow outcome score".ab,ti. |
| 7 | "Rancho Los Amigos Scale".ab,ti. |
| 8 | exp unconsciousness/ or exp coma/ |
| 9 | (Unconscious$ or coma$ or concuss$ or 'persistent vegetative state').ab,ti. |
| 10 | ((head or crani$ or cerebr$ or capitis or brain$ or forebrain$ or skull$ or hemispher$ or intra-cran$ or inter-cran$) adj3 (injur$ or trauma $ or damag$ or wound$ or fracture$ or contusion$)).ab,ti. |
| 11 | "Diffuse axonal injur$".ab,ti. |
| 12 | ((head or crani$ or cerebr$ or brain$ or intra-cran$ or inter-cran$) adj3 (haematoma$ or hematoma$ or haemorrhag$ or hemorrhag $ or bleed$ or pressure)).ab,ti. |
| 13 | exp glasgow coma scale/ or exp glasgow outcome scale/ or exp rancho los amigos scale/ |
| 14 | or/4-13 |
| 15 | 1 or 2 or 3 or 14 |
| 16 | systematic* review*.tw. |
| 17 | meta-analysis as topic/ |
| 18 | (meta-analytic* or meta-analysis or metanalysis or metaanalysis or meta analysis or meta-synthesis or metasynthesis or meta synthesis or meta-regression or metaregression or meta regression).tw. |
| 19 | (synthes* adj3 literature).tw. |
| 20 | (synthes* adj3 evidence).tw. |
| 21 | (integrative review or data synthesis).tw. |
| 22 | (research synthesis or narrative synthesis).tw. |
| 23 | (systematic study or systematic studies).tw. |
| 24 | (systematic comparison* or systematic overview*).tw. |
| 25 | ((evidence based or comprehensive or critical or quantitative or structured) adj review).tw. |
| 26 | (realist adj (review or synthesis)).tw. |
| 27 | or/16-26 |
| 28 | ((animal or nonhuman) not (human and (animal or nonhuman))).de. |
| 29 | (27 not 28) {Including Related Terms} |
| 30 | 14 and 29 |
| 31 | exp complication$/ |
| 32 | Exp adverse event$/ |
| 33 | adverse event$ {Including Related Terms} |
| 34 | “complicity”.ab.ti. |
| 35 | 31 or 32 or 33 or 34 |
| 36 | 30 and 35 |
