# Supplementary material for: Overview of systematic reviews: Management of common Traumatic Brain Injury-related complications
Source: PLoS One. 2022 Sep 1;17(9):e0273998. doi: 10.1371/journal.pone.0273998 (PMC9436148; doi:10.1371/journal.pone.0273998)
Supplement: S3 Appendix — (DOCX) [file pone.0273998.s003.docx]

**S 3 Appendix. Medline searched strategy**

1 exp head injury/

2 exp Cerebrovascular Accident/

3 exp Brain Edema/

4 exp Glasgow coma scale/

5 exp Glasgow outcome scale/

6 exp Glasgow outcome score/

7 exp Rancho Los Amigos Scale/

8 exp unconsciousness/ or exp coma/

9 (Unconscious$ or coma$ or concuss$ or 'persistent vegetative state').ab,ti.

10 ((head or crani$ or cerebr$ or capitis or brain$ or forebrain$ or skull$ or hemispher$ or intra-cran$ or inter-cran$) adj3 (injur$ or trauma $ or damag$ or wound$ or fracture$ or contusion$)).ab,ti.

11 "Diffuse axonal injur$".ab,ti.

12 ((head or crani$ or cerebr$ or brain$ or intra-cran$ or inter-cran$) adj3 (haematoma$ or hematoma$ or haemorrhag$ or hemorrhag $ or bleed$ or pressure)).ab,ti.

13 (glasgow coma scale or glasgow outcome scale or rancho los amigos scale).ab,ti.

14 or/4-13

15 1 or 2 or 3 or 14

16 systematic* review*.tw.

17 meta-analysis as topic/

18 (meta-analytic* or meta-analysis or metanalysis or metaanalysis or meta analysis or meta-synthesis or metasynthesis or meta synthesis or meta-regression or metaregression or meta regression).tw.

19 (synthes* adj3 literature).tw.

20 (synthes* adj3 evidence).tw.

21 (integrative review or data synthesis).tw.

22 (research synthesis or narrative synthesis).tw.

23 (systematic study or systematic studies).tw.

24 ((systematic adj (review$1 or overview$1)).tw.

25 ((evidence based or comprehensive or critical or quantitative or structured) adj review).tw.

26 exp Review Literature as Topic/

27 or/16-26

28 ((animal or nonhuman) not (human and (animal or nonhuman))).de.

29 (27 not 28)

30 14 and 29

31 exp complication$/

32 exp adverse event$/

33 exp adverse event$

34 “complicity”.ab.ti.

35 or/31-34

36 30 and 35
