# Supplementary material for: Overview of systematic reviews: Management of common Traumatic Brain Injury-related complications
Source: PLoS One. 2022 Sep 1;17(9):e0273998. doi: 10.1371/journal.pone.0273998 (PMC9436148; doi:10.1371/journal.pone.0273998)
Supplement: S4 Appendix — (DOCX) [file pone.0273998.s004.docx]

**S4 Appendix. Quality assessment (R-AMSTAR) of included systematic reviews**

| **R-AMSTAR**  **Criteria^*^** | **Author, year** | | | | | |
| --- | --- | --- | --- | --- | --- | --- |
|  | **Hassett 2017** | **Synnot 2017** | **Thompson 2015** | **Bakr 2018** | **Meshkini 2015** | **Khan**  **2016** |
| **1. Was an a priori design provided?** | | | | | | |
| A. Clearly focussed (PICO-based) question | Y | Y | Y | Y | Y | Y |
| B. Description of inclusion criteria | Y | Y | Y | Y | Y | Y |
| C. Study protocol published, registered in advance, or both | Y | Y | Y | Y | N | N |
| **SCORE** | **4** | **4** | **4** | **4** | **3** | **3** |
| **2. Was there duplicate study selection and data extraction?** | | | | | | |
| A. ­≥2 persons independently extracted the data, explicitly stated | Y | Y | Y | N | Y | Y |
| B. Statement of consensus procedure for disagreements | Y | Y | Y | N | Y | Y |
| C. Disagreements among extractors resolved properly | Y | Y | Y | N | Y | Y |
| **SCORE 4 4 4 1 4 4** | | | | | | |
| **3. Was a comprehensive literature search performed?** | | | | | | |
| A. At least two electronic sources are searched | Y | Y | Y | Y | Y | Y |
| B. Years & databases used are mentioned | Y | Y | Y | Y | Y | Y |
| C. Key words, MeSH terms (or both) C. Key words, MeSH terms (or both) are stated, and where feasible, the search strategy outline is provided | Y | Y | Y | Y | Y | Y |
| D. Searches are supplemented by consulting current contents, reviews, textbooks, registers, and by reviewing the references in the studies found | Y | Y | Y | N | N | N |
| E. Journals are hand searched or manual searched | Y | Y | Y | N | N | N |
| **SCORE 4 4 4 3 3 3** | | | | | | |
| **4. Was the status of publication**  **(i.e. grey literature) used as an inclusion criterion?** | | | | | | |
| A. Searched for reports regardless of their publication type | Y | Y | Y | N | N | N |
| B. Any reports based on their publication status, language, etc. excluded | Y | Y | Y | Y | Y | Y |
| C. Non-English papers were translated, or readers sufficiently trained in foreign language | Y | Y | Y | N | N | N |
| D. No language restriction or recognition of non-English articles | Y | Y | Y | N | N | N |
| **SCORE 4 4 4 2 2 2** | | | | | | |
| **5. Was a list of studies (included and excluded) provided?** | | | | | | |
| A. Table, list, figure of included studies; a reference list does not suffice | Y | Y | Y | Y | Y | Y |
| B. Table, list, figure of excluded studies either in the article or in a supplemental source | Y | Y | Y | N | N | N |
| C. Satisfactory/ sufficient statement of the reason for exclusion of the seriously considered studies | Y | Y | Y | Y | N | Y |
| D. Reader is able to retrace the included and the excluded studies anywhere in the article bibliography, reference, or supplemental source | Y | Y | Y | N | N | N |
| **SCORE 4 4 4 2 1 2** | | | | | | |
| **6. Were the characteristics of the included studies provided?** | | | | | | |
| A. Participants, interventions, exposure, and outcomes from the original studies provided in an aggregated form | Y | Y | Y | Y | Y | Y |
| B. Ranges are provided of the relevant characteristics in the studies analysed | Y | Y | Y | Y | Y | Y |
| C. Information provided appears to be complete and accurate | Y | Y | Y | Y | Y | Y |
| **SCORE 4 4 4 4 4 4** | | | | | | |
| **7. Was the scientific quality of the included studies assessed and documented?** | | | | | | |
| A. A priori methods are provided | Y | Y | Y | Y | Y | Y |
| B. Scientific quality of the included studies appears to be meaningful | Y | Y | Y | Y | Y | Y |
| C. Discussion, recognition, awareness of level of evidence is present | Y | Y | Y | Y | Y | Y |
| D. Quality of evidence rated, ranked, based on characterised instruments | Y | Y | Y | Y | Y | Y |
| **SCORE 4 4 4 4 4 4** | | | | | | |
| **8. Was the scientific quality of the included studies used appropriately in formulating conclusions?** | | | | | | |
| A. Scientific quality considered in the analysis and the conclusions of the review | Y | Y | Y | Y | Y | Y |
| B. Scientific quality is explicitly stated in formulating recommendations | Y | Y | Y | Y | Y | Y |
| C. Conclusions integrated, drives towards practice guidelines | Y | Y | Y | Y | Y | Y |
| D. Clinical consensus statement drives toward revision or confirmation of practice guidelines | Y | Y | Y | Y | N | Y |
| **SCORE** | **4** | **4** | **4** | **4** | **3** | **4** |
| **9. Were the methods used to combine the findings of studies appropriate?** | | | | | | |
| A. Statement of criteria that were used to decide that the studies analysed were similar enough to be pooled | Y | Y | Y | Y | Y | Y |
| B. For the pooled results, attest is done to ensure the studies were combinable, to assess their homogeneity | Y | Y | Y | Y | Y | Y |
| C. A recognition of heterogeneity or lack of thereof is present | Y | Y | Y | N | Y | Y |
| D. If heterogeneity exists, a random-effects model is used, the rationale of combining is taken into consideration, or both | Y | Y | Y | N | Y | Y |
| E. If homogeneity exists, a rationale or a statistical test is stated | Y | Y | Y | N | Y | Y |
| **SCORE 4 4 4 2 4 4** | | | | | | |
| **10. Was the likelihood of publication bias assessed?** | | | | | | |
| A. Recognition of publication bias or file drawer effect | Y | Y | Y | N | Y | Y |
| B. Graphical aids (e.g. funnel plot) | N | N | N | N | Y | Y |
| C. Statistical tests (e.g. Egger regression test) | Y | Y | Y | N | Y | N |
| **SCORE 3 3 3 1 4 3** | | | | | | |
| **11. Was the conflict of interest included?** | | | | | | |
| A. Statement of sources of support | Y | Y | Y | Y | Y | Y |
| B. No conflict of interest | Y | Y | Y | Y | Y | Y |
| C. An awareness, statement of support or conflict of interest in the primary inclusion studies | N | N | N | N | N | N |
| **SCORE 3 3 3 3 3 3** | | | | | | |
| **TOTAL SCORE (Out of 44) 42 42 42 30 35 36** | | | | | | |
