# Supplementary material for: Overview of systematic reviews: Management of common Traumatic Brain Injury-related complications
Source: PLoS One. 2022 Sep 1;17(9):e0273998. doi: 10.1371/journal.pone.0273998 (PMC9436148; doi:10.1371/journal.pone.0273998)
Supplement: S5 Appendix — (DOCX) [file pone.0273998.s005.docx]

**S5 Appendix. Details of excluded reviews**

| **Ref no.** | **Author and year** | **Articles excluded with reason** |
| --- | --- | --- |
| 1 | Coggrave et al, 2014 | TBI specific sub group data not provided |
| 2 | Barrera et al, 2013 | Data not consistent with TBI |
| 3 | Mollayeva et al, 2013 | Not an interventional review |
| 4 | Ponsford et al, 2012 | Not systematic review |
| 5 | Tan et al, 2017 | Clinical practice guidelines |
| 6 | Richmond, 2014 | Narrative review |
| 7 | Paterniti et al, 2015 | Narrative review |
| 8 | Mesquita et al, 2010 | Not systematic review |
| 9 | Kreitschmann-Andermahr et al, 2011 | Not systematic review |
| 10 | Hohl et al, 2009 | Narrative review |
| 11 | Xu et al, 2017 | Not an interventional review |
| 12 | Pinder et al, 2011 | Case reports |
| 13 | Kaimovskii et al, 2013 | Narrative review |
| 14 | Billiard et al, 2013 | Case reports |
| 15 | Bell et al, 2018 | Narrative review |
| 16 | Sampathkumar et al, 2018 | Not an interventional review |
| 17 | Vermaelen et al, 2015 | Not systematic review |
| 18 | Mollayeva et al, 2014 | Not an interventional review |
| 19 | Larson et al, 2018 | Not systematic review |
| 20 | Regan et al, 2014 | TBI specific sub group data not provided |
| 21 | Almangour et al, 2016 | Not an interventional review |
| 22 | Chaari et al, 2017 | TBI specific sub group data not provided |
| 23 | Yang et al, 2016 | Data not specific for adult population |
| 24 | Zhao et al, 2018 | TBI specific sub group data not provided |
| 25 | Wilson et al, 2018 | Does not provide age specific data |
| 26 | Zafar et al, 2012 | TBI specific sub group data not provided |
| 27 | Xu et al, 2016 | Data not specific for adult population |
| 28 | Grima et al, 2016 | Not an interventional review |
| 29 | Wat et al, 2019 | Data not specific for adult population |
