# Supplementary material for: Overview of systematic reviews: Management of common Traumatic Brain Injury-related complications
Source: PLoS One. 2022 Sep 1;17(9):e0273998. doi: 10.1371/journal.pone.0273998 (PMC9436148; doi:10.1371/journal.pone.0273998)
Supplement: S6 Appendix — (DOCX) [file pone.0273998.s006.docx]

**S 6 Appendix. References to studies excluded from this review**

1. Coggrave M, Norton C, Cody JD. Management of faecal incontinence and constipation in adults with central neurological diseases. Cochrane Database Syst Rev. 2014(1); CD002115.

2. Barrera LM, Perel P, Ker K, Cirocchi R, Farinella E, Morales Uribe CH. Thromboprophylaxis for trauma patients. Cochrane Database Syst Rev. 2013 (3); CD008303.

3. Mollayeva T, Colantonio A, Mollayeva S, Shapiro CM. Screening for sleep dysfunction after traumatic brain injury. Sleep Med. 2013;14(12):1235-46.

4. Ponsford JL, Ziino C, Parcell DL, Shekleton JA, Roper M, Redman JR et al. Fatigue and sleep disturbance following traumatic brain injury--their nature, causes, and potential treatments. J Head Trauma Rehabil. 2012;27 (3):224-33.

5. Tan CL, Alavi SA, Baldeweg SE, Belli A, Carson A, Feeney C et al. The screening and management of pituitary dysfunction following traumatic brain injury in adults: British Neurotrauma Group guidance. J Neurol Neurosurg Psychiatry. 2017 Nov;88(11):971-81.

6. Richmond E, Rogol AD. Traumatic brain injury: endocrine consequences in children and adults. Endocrine. 2014;45(1):3-8.

7. Paterniti I, Cordaro M, Navarra M, Esposito E, Cuzzocrea S. Emerging pharmacotherapy for treatment of traumatic brain injury: targeting hypopituitarism and inflammation. Expert Opin Emerg Drugs. 2015;20(4):583-96.

8. Mesquita J, Varela A, Medina JL. Trauma and the endocrine system. Endocrinol Nutr. 2010;57(10):492-99.

9. Kreitschmann-Andermahr I, Brabant G. Neuroendocrine disturbances after acquired brain damage. Fortschritte der Neurologie-Psychiatrie. 2011;79(4):213-20.

10. Hohl A, Mazzuco TL, Coral MH, Schwarzbold M. Hypogonadism after traumatic brain injury. Arquivos brasileiros de endocrinologia e metabologia. 2009;53(8):908-14.

11. Xu T, Yu X, Ou S, Liu X, Yuan J, Huang H, et al.. Risk factors for posttraumatic epilepsy: A systematic review and meta-analysis. Epilepsy Behav. 2017; 67:1-6.

12. Pinder C, Young C. Adverse cognitive effects of phenytoin in severe brain injury: a case report. Brain Inj. 2011;25(6):634-37.

13. Kaimovskii IL, Lebedeva AV, Mutaeva T, Gorshkov KM, Krylov VV, Talypov et al. Risk factors for posttraumatic epilepsy in adults. Zh Nevrol Psikhiatr Im S S Korsakova 2013; 113:25-28.

14. Billiard M, Podesta C. Recurrent hypersomnia following traumatic brain injury. Sleep Med. 2013;14(5):462-65.

15. Bell KR, Bushnik T, Dams-O'Connor K, Goldin Y, Hoffman JM, Lequerica AH et al. Sleep after TBI: How the TBI Model Systems have advanced the field. NeuroRehabilitation. 2018;43(3):287-96.

16. Sampathkumar H, DiTommaso C, Holcomb E, Tallavajhula S. Assessment of sleep after traumatic brain injury (TBI). NeuroRehabilitation. 2018;43(3):267-76.

17. Vermaelen J, Greiffenstein P, deBoisblanc BP. Sleep in traumatic brain injury. Crit Care Clin. 2015;31(3):551-61.

18. Mollayeva T, Kendzerska T, Mollayeva S, Shapiro CM, Colantonio A, Cassidy JD. A systematic review of fatigue in patients with traumatic brain injury: The course, predictors and consequences. Neurosci Biobehav Rev. 2014; 47:684-716.

19. Larson EB. Sleep disturbance and cognition in people with TBI. NeuroRehabilitation. 2018;43(3):297-306.

20. Regan J, Murphy A, Chiang M, McMahon BP, Coughlan T, Walshe M. Botulinum toxin for upper oesophageal sphincter dysfunction in neurological swallowing disorders. Cochrane Database Syst Rev 2014 (5); CD0099968.

21. Almangour W, Schnitzler A, Salga M, Debaud C, Denormandie P, Genêt F. Recurrence of heterotopic ossification after removal in patients with traumatic brain injury: A systematic review. Ann Phys Rehabil Med. 2016;59(4):263-9.

22. Chaari A, Mohamed AS, Abdelhakim K, Kauts V, Casey WF. Levetiracetam versus phenytoin for seizure prophylaxis in brain injured patients: a systematic review and meta-analysis. Int J Clin Pharm. 2017;39(5):998-1003.

23. Yang Y, Zheng F, Xu X, Wang X. Levetiracetam Versus Phenytoin for Seizure Prophylaxis Following Traumatic Brain Injury: A Systematic Review and Meta-Analysis. CNS drugs. 2016;30(8):677-88.

24. Zhao L, Wu YP, Qi JL, Liu YQ, Zhang K, Li WL. Efficacy of levetiracetam compared with phenytoin in prevention of seizures in brain injured patients: A meta-analysis. Medicine (Baltimore). 2018;97(48):e13247.

25. Wilson CD, Burks JD, Rodgers RB, Evans RM, Bakare AA, Safavi-Abbasi S. Early and Late Posttraumatic Epilepsy in the Setting of Traumatic Brain Injury: A Meta-analysis and Review of Antiepileptic Management. World Neurosurg. 2018;110: e901-e906.

26. Zafar SN, Khan AA, Ghauri AA, Shamim MS. Phenytoin versus Levetiracetam for seizure prophylaxis after brain injury - a meta-analysis. BMC Neurol. 2012; 12:30.

27. Xu JC, Shen J, Shao WZ, Tang LJ, Sun YZ, Zhai XF et al. The safety and efficacy of levetiracetam versus phenytoin for seizure prophylaxis after traumatic brain injury: A systematic review and meta-analysis. Brain Inj. 2016;30(9):1054-61.

28. Grima N, Ponsford J, Rajaratnam SM, Mansfield D, Pase MP. Sleep Disturbances in Traumatic Brain Injury: A Meta-Analysis. J Clin Sleep Med. 2016;12(3):419-28.

29. Wat R, Mammi M, Paredes J, Haines J, Alasmari M, Liew A et al. The Effectiveness of Antiepileptic Medications as Prophylaxis of Early Seizure in Patients with Traumatic Brain Injury Compared with Placebo or No Treatment: A Systematic Review and Meta-Analysis. World Neurosurg. 2019;122:433-40.
